# SGLT2 Inhibitors in Alzheimer’s Disease: Biochemical Insights and Therapeutic Potential

**DOI:** 10.3390/ijms27115051

**Published:** 2026-06-03

**Authors:** Pietro Mazzeo, Mariapia Vietri, Nicola Tecce, Alessia Delizia, Valentina Remondelli, Tania Ciaglia, Anna Di Dio, Laura Corletti, Carmine Gerardo Pizzuti, Giacomo Pepe, Eugenio Stabile, Michele Correale, Pietro Campiglia, Maria Rosaria Miranda, Mario Felice Tecce, Vincenzo Vestuto

**Affiliations:** 1 Department of Pharmacy, University of Salerno, Via G. Paolo II, 84084 Fisciano, Italy; pmazzeo@unisa.it (P.M.); mvietri@unisa.it (M.V.); alessia.delizia@libero.it (A.D.); tciaglia@unisa.it (T.C.); a.didio6@studenti.unisa.it (A.D.D.); l.corletti@studenti.unisa.it (L.C.); c.pizzuti@studenti.unisa.it (C.G.P.); gipepe@unisa.it (G.P.); pcampiglia@unisa.it (P.C.); tecce@unisa.it (M.F.T.); vvestuto@unisa.it (V.V.); 2 Division of Cardiology, Cardiovascular Department, Azienda Ospedaliera Regionale “San Carlo”, 85100 Potenza, Italy; eugeniostabile@gmail.com; 3 Endocrinology Unit, Department of Clinical Medicine and Surgery, Federico II University, 80138 Naples, Italy; nicola.tecce@unina.it; 4 Department of Psychology, University of Campania “Luigi Vanvitelli”, 81100 Caserta, Italy; vremondelli@hotmail.com; 5 National Biodiversity Future Center (NBFC), 90133 Palermo, Italy; 6 Department of Health Sciences, University of Basilicata, 85100 Potenza, Italy; 7 Emergency and Urgency Department, Ospedali Riuniti University Hospital, 71100 Foggia, Italy; 8 Department of Medicine, Digestive Health Research Institute, Case Western Reserve University School of Medicine, Cleveland, OH 44106, USA

**Keywords:** SGLT2 inhibitors, Alzheimer’s disease, metabolic remodeling, neurodegenerative therapy, preclinical and clinical studies

## Abstract

Sodium–glucose cotransporter-2 (SGLT2) inhibitors, initially developed as antidiabetic agents, have recently gained attention for their potential role in modulating processes relevant to Alzheimer’s disease (AD). Preclinical studies suggest that they may influence key mechanisms involved in AD. However, available clinical studies, mainly retrospective and focused on diabetic populations, provide insufficient clarity on whether these effects extend to broader, non-diabetic groups. The heterogeneity of neurodegenerative diseases, which differ in inflammatory and proteotoxic mechanisms, further highlights the need for disease-specific investigations. This review examines mechanistic pathways through which SGLT2 inhibition may influence AD progression and evaluates current clinical evidence, aiming to identify key knowledge gaps and guide future research. This review summarises the latest evidence from the literature, focusing on preclinical experiments, translational studies and early clinical observations. The search focused on pathways related to microglial and astrocytic activation, oxidative stress, metabolic remodeling, neuronal survival, and amyloid and tau dynamics. Accumulating data indicate that SGLT2 inhibitors exert multifaceted actions relevant to AD pathology, including reduced neuroinflammation and oxidative stress, improved mitochondrial and insulin signaling, as well as decreased amyloid deposition and tau hyperphosphorylation. Additionally, SGLT2 inhibition may improve cerebrovascular perfusion and blood–brain barrier stability, potentially supporting cognitive function. Nonetheless, major challenges remain, including variable blood–brain barrier permeability and heterogeneous experimental responses. SGLT2 inhibitors represent a promising pleiotropic class of compounds with potential disease-modifying effects in AD. Their capacity to target metabolic, inflammatory, and proteotoxic pathways makes them attractive candidates for neurodegenerative therapy. Further studies are required to clarify biochemical pathways and validate clinical efficacy.

## 1. Introduction

The global burden of neurodegenerative diseases, particularly Alzheimer’s disease (AD), is rising in parallel with metabolic disorders such as type 2 diabetes mellitus (T2DM) [1]. Chronic hyperglycemia, insulin resistance, and sustained metabolic stress, collectively termed glucotoxicity, are recognized as central contributors to neuronal dysfunction, synaptic loss, blood–brain barrier (BBB) disruption, and neuroinflammation. Beyond systemic complications, metabolic dysregulation profoundly reshapes cerebral bioenergetics and redox homeostasis, thereby accelerating age-related cognitive decline [2].

Growing evidence conceptualizes AD as a state of brain insulin resistance, or “type 3 diabetes”, where impaired insulin signaling promotes tau hyperphosphorylation, amyloidogenic processing and mitochondrial failure [2,3]. Concomitantly, chronic metabolic stress activates inflammatory sensors, further amplifying microglial reactivity and oxidative injury. Together, these events establish a self-reinforcing metabolic–inflammatory–proteotoxic loop that drives neurodegeneration [4].

While traditional antidiabetic therapies focus on glycemic control, odium–glucose cotransporter 2 (SGLT2) inhibitors, commonly known as “gliflozins”, exert pleiotropic effects that may intersect with these pathogenic pathways. Originally developed to promote glycosuria and reduce plasma glucose via blockade of renal proximal-tubule SGLT2, these agents (empagliflozin, dapagliflozin, canagliflozin, etc.) demonstrate systemic benefits in cardiovascular and renal health. Intriguingly, preclinical and emerging clinical data support a potential role of SGLT2 inhibitors (SGLT2i) in ameliorating neurodegenerative processes [5,6,7].

These lipophilic agents can cross the blood–brain barrier (BBB), although SGLT2 expression in the brain remains extremely low and localized [8].

The potential neuroprotective role of SGLT2 inhibitors may arise from systemic metabolic remodeling, such as enhanced ketogenesis and reduced inflammatory load or direct central actions. Epidemiological data suggest that T2DM patients treated with SGLT2 inhibitors have a lower incidence of dementia compared to those on other glucose-lowering therapies. Moreover, preclinical in vitro studies in neuronal cultures and in vivo in rodent models of neurodegeneration, including AD models, demonstrate that gliflozins mitigate Aβ-induced neurotoxicity, reduce tau phosphorylation, suppress neuroinflammation, protect neuronal morphology and synaptic markers, and improve cognitive or behavioural outcomes [9,10,11].

Despite these encouraging findings, major gaps remain: the central nervous system distribution of SGLT2i, their direct effects on neuronal vs. glial cells, the relative contribution of improved systemic metabolism versus local neuroprotective mechanisms, and long-term safety in non-diabetic individuals. As such, a comprehensive synthesis of biochemical insights, preclinical data, and emerging clinical evidence is timely and essential to guide future translational efforts.

In this review, we provide a detailed analysis of the neuroprotective mechanisms of SGLT2 inhibitors: contextualizing glucotoxicity as a trigger of neurodegeneration; outlining the cellular and molecular pathways modulated by SGLT2i (oxidative stress, mitochondrial function, autophagy, inflammation, vascular and blood–brain barrier (BBB) integrity); summarizing in vitro and in vivo evidence from models of neurodegeneration; focusing on Alzheimer’s disease as a paradigmatic disorder likely to benefit; and discussing translational challenges and future directions for clinical application.

## 2. SGLT2 Inhibitors: From Glucose Control to Systemic Metabolic and Cardiovascular Effects

SGLT2i, also known as gliflozins, inhibit renal glucose reabsorption by blocking sodium–glucose co-transporters located in the S1 segment of the proximal convoluted tubules, thereby inducing osmotic diuresis. This mechanism results in glycosuria, improved glycemic control, and maintenance of glucose homeostasis. In addition, inhibition of the SGLT2 isoform reduces intraglomerular pressure and contributes to improved blood pressure control through a sustained negative sodium–water balance [12].

SGLT2i are among the most recently introduced drugs in the treatment of type 2 diabetes mellitus (T2DM), and their beneficial effects, when added to standard care, on renal protection, cardiovascular protection, and heart failure outcomes have been remarkable in primary clinical studies [13]. Beyond their established cardioprotective and nephroprotective properties, SGLT2i exert multiple pleiotropic actions, influencing blood pressure levels, body weight, lipid and uric acid metabolism, haematocrit, and hepatic steatosis (Table 1) [14].

Recent placebo-controlled clinical trials have demonstrated that SGLT2 inhibition reduces cardiovascular death (CD), progression of chronic kidney disease (CKD), and all-cause mortality in both diabetic and non-diabetic patients. Although the precise mechanisms underlying these benefits are not fully elucidated, accumulating preclinical and clinical evidence suggests that SGLT2i play an important role in stabilizing cellular metabolic stress, attenuating glucose-mediated toxicity, and reducing inflammation in key organs such as the kidneys and heart [15,16].

At the metabolic level, inhibition of the SGLT2 isoform induces a shift in glucose metabolism characterized by increased fat oxidation and ketogenesis, together with reduced carbohydrate utilization (Table 1) [17]. This metabolic remodeling is associated with an average body weight reduction of approximately 2 kg over 3–6 months [18], driven by decreased visceral and subcutaneous fat mass, reduced waist circumference, and improved insulin sensitivity.

From a cardiorenal perspective, SGLT2 inhibition not only prevents proximal glucose reabsorption but also blocks proximal sodium reabsorption, leading to increased sodium delivery to the macula densa, natriuresis, and osmotic diuresis [19]. The initial increase in sodium excretion produces an approximate 7% reduction in plasma volume. On one hand, this contributes to reduced hospitalization rates for heart failure, as demonstrated in several recent clinical trials; on the other hand, the sustained increase in fractional sodium excretion is associated with significant reductions in blood pressure (Table 1) [20,21,22,23]. Given that most patients with CKD exhibit sodium-sensitive hypertension, the natriuretic effect of SGLT2 inhibition may exert an additional antihypertensive benefit in this population [24].

In 2008, the United States Food and Drug Administration (FDA) introduced specific regulatory guidance requiring that new antihyperglycemic agents demonstrate no increased risk of myocardial infarction, stroke, or major cardiovascular events through dedicated cardiovascular outcome trials (CVOTs). Within this regulatory framework, four SGLT2 inhibitors—canagliflozin, dapagliflozin, empagliflozin, and ertugliflozin [25]—were approved, with sotagliflozin more recently receiving FDA approval in 2023.

Three of these agents were subsequently evaluated in large randomized controlled trials that proved pivotal in defining the cardiovascular profile of the class: EMPA-REG OUTCOME, CANVAS, and DECLARE–TIMI 58. These landmark studies demonstrated significant reductions in cardiovascular risk and macrovascular complications in patients with type 2 diabetes treated with empagliflozin, canagliflozin, and dapagliflozin, respectively. In both EMPA-REG OUTCOME and CANVAS, the primary endpoint was the traditional three-point composite of major adverse cardiovascular events (MACE), defined as time to first occurrence of non-fatal myocardial infarction, non-fatal stroke, or cardiovascular death (Table 2) [24].

Importantly, diabetes mellitus shares several pathophysiological mechanisms with neurodegenerative disorders, including systemic inflammation, oxidative stress, insulin resistance, and advanced glycation end-product formation [26]. In this context, the systemic metabolic and vascular effects of SGLT2 inhibition have prompted growing interest in their potential role beyond the cardio–renal axis, including possible neuroprotective implications in cognitive decline and Alzheimer’s disease [27].

**Table 1:** Systemic metabolic effects of SGLT2 inhibition.

Biological Effect	Mechanism	Systemic Consequence	References
Glycosuria	Inhibition of renal SGLT2 transporters in the proximal tubule	Reduced plasma glucose and improved glycemic control	[12]
Natriuresis	Reduced proximal sodium reabsorption	Decreased plasma volume and blood pressure	[19,20,21,22]
Metabolic shift toward fat oxidation and ketogenesis	Reduced glucose availability promotes fatty acid utilization	Increased ketone body production and metabolic remodeling	[17]
Body weight reduction	Increased glycosuria and metabolic shift	Decreased visceral and subcutaneous fat mass and improved insulin sensitivity	[18]
Anti-inflammatory effects	Reduction of glucose-mediated metabolic stress	Attenuation of systemic inflammatory signaling	[15,16]
Reduction of oxidative stress	Improved metabolic efficiency and reduced glucotoxicity	Stabilization of cellular redox balance	[15,16]
Improved endothelial and vascular function	Natriuresis and reduced intraglomerular pressure	Improved blood pressure control and vascular homeostasis	[12,19,20,21,22]

**Table 2:** Major clinical trials evaluating the effects of SGLT2 inhibitors.

Trial	Drug	Population	Main Cardiovascular Outcomes	Renal Outcomes	Key Findings	Ref.
EMPA-REG OUTCOME	Empagliflozin	T2DM + established cardiovascular disease	Significant reduction in CV death and hospitalization for heart failure	Reduced renal composite outcome (doubling creatinine, RRT, renal death)	First major trial demonstrating strong cardiovascular and renal protection with SGLT2 inhibition	[24,28,29]
CANVAS Program	Canagliflozin	T2DM with established CVD or high CV risk	Reduction in hospitalization for heart failure	Reduced progression of albuminuria and composite renal outcomes	Confirmed cardiovascular benefit of SGLT2 inhibition in high-risk patients	[24,30,31,32,33]
DECLARE–TIMI 58	Dapagliflozin	T2DM with or without established CVD	Significant reduction in hospitalization for heart failure	Reduced renal composite endpoint	Largest SGLT2 inhibitor CVOT; demonstrated strong HF and renal benefits	[34]
DAPA-CKD	Dapagliflozin	CKD patients with or without diabetes	Reduction in hospitalization for HF or CV death	Strong reduction in kidney disease progression	Demonstrated renoprotective effects independent of diabetes	[34,35,36]
EMPA-KIDNEY	Empagliflozin	CKD population including non-diabetic patients	Reduction in CV death or hospitalization for HF	Reduced kidney disease progression	Confirmed renal protection in broader CKD population	[13,35,37]
DAPA-HF	Dapagliflozin	Heart failure with reduced ejection fraction (HFrEF)	Reduced HF hospitalization and CV death	Slower decline in eGFR	Demonstrated benefit in HF independent of diabetes status	[38]
DELIVER	Dapagliflozin	HF with mildly reduced or preserved EF (HFmrEF/HFpEF)	Reduced HF events across EF spectrum	—	Extended SGLT2 inhibitor benefit to HFpEF populations	[39]
EMPEROR-Reduced/EMPEROR-Preserved	Empagliflozin	HFrEF and HFpEF populations	Reduced HF hospitalization	Slowed renal function decline	Confirmed benefit across heart failure phenotypes	[40,41]

### 2.1. Clinical Validation of SGLT2 Inhibition

In the EMPA-REG OUTCOME study, a multicentre, randomized, double-blind, placebo-controlled trial involving 7020 patients with type 2 diabetes and established cardiovascular disease, empagliflozin significantly reduced major adverse cardiovascular events and cardiovascular mortality (*p* < 0.001), whilst no significant differences were observed with regard to non-fatal myocardial infarction or stroke. Hospitalization for heart failure (HHF) or cardiovascular death (excluding fatal strokes) occurred in 5.7% of patients treated with empagliflozin compared with 8.5% in the placebo group (*p* < 0.001). In addition to cardiovascular benefits, empagliflozin significantly improved renal outcomes [24,25,26,27,28]. Furthermore, patients with T2DM at high cardiovascular risk treated with empagliflozin showed lower rates of the primary composite cardiovascular outcome and all-cause mortality when the drug was added to standard therapy [29]. These findings were subsequently reinforced by the CANVAS Program, a multicenter, randomized, double-blind trial including 10,142 patients with T2DM, approximately two-thirds of whom had established cardiovascular disease [24,30,31]. As in EMPA-REG OUTCOME, a marked reduction was observed in the secondary endpoint of hospitalization for heart failure, and the rate of HHF or cardiovascular death per 1000 patient-years was 16.3 in the canagliflozin group compared to 20.8 in the placebo group (*p* = 0.0015) [30,32]. Furthermore, CANVAS demonstrated a 25% reduction in progression of albuminuria and a 40% reduction in the composite renal outcome of ≥40% reduction in estimated glomerular filtration rate (eGFR), RRT, or renal death (Table 2) [33].

The DECLARE–TIMI 58 trial, the largest SGLT2 inhibitor CVOT, enrolled 17,160 patients with T2DM and randomized them to dapagliflozin (10 mg daily) or placebo over a median follow-up of 4.2 years. The primary composite cardiovascular endpoint occurred in 8.8% of dapagliflozin-treated patients compared to 9.4% of placebo patients (*p* = 0.17). However, significant reductions were observed in hospitalization for heart failure (2.5% vs. 3.3%; *p* = 0.0008) and in the composite of HHF or cardiovascular death (4.9% vs. 5.8%; *p* = 0.005) (Table 2). Renal outcomes were also favourably affected, occurring in 1.5% of dapagliflozin patients compared to 2.8% in the placebo group (*p* < 0.001). Thus, although dapagliflozin did not significantly reduce MACE in this broader population, it significantly reduced hospitalization for heart failure and improved renal outcomes [34].

Based on these findings, the DAPA-CKD study demonstrated that the nephroprotective effects of SGLT2 inhibition extend to patients with chronic kidney disease, regardless of whether or not they have diabetes. In this study, dapagliflozin significantly reduced the risk of end-stage kidney disease or death from renal or cardiovascular causes, consistent with the previous findings of the DECLARE–TIMI 58 study (Table 2) [34,35,36].

Similarly, the EMPA-KIDNEY study demonstrated that empagliflozin consistently reduced the progression of kidney disease or death from cardiovascular causes (Table 2) [13,35,37]. In the DAPA-HF study, dapagliflozin significantly reduced events of worsening heart failure and cardiovascular mortality in patients with heart failure with reduced ejection fraction (HFrEF), regardless of baseline diabetic status (Table 2) [38]. Importantly, baseline renal function did not modify the observed benefit, and dapagliflozin slowed the rate of eGFR decline even in non-diabetic individuals. The DELIVER study extended this evaluation to patients with left ventricular ejection fraction (LVEF) >40%, including both HFpEF and HF with mildly reduced ejection fraction (HFmrEF) (Table 2). Together with DAPA-HF, DELIVER provided evidence supporting dapagliflozin efficacy across the full spectrum of LVEF [39].

In parallel, the EMPEROR-Reduced and EMPEROR-Preserved trials assessed empagliflozin in patients with HFrEF and HFpEF, respectively, with or without diabetes. Real-world and observational studies have further contributed to the systemic validation of SGLT2 inhibition, such as the prospective observational study by Loria et al. evaluated the clinical, biochemical, echocardiographic, and functional impact of dapagliflozin in patients with HFrEF under different hemodynamic conditions [40]. Furthermore, the ongoing international multicenter BEGIN-HF registry is designed to further characterize changes in biventricular function and clinical parameters in patients initiating SGLT2 inhibitors in routine practice (Table 2) [41].

Collectively, randomized cardiovascular and renal outcome trials, dedicated heart failure studies, and real-world evidence through the reproducibility of cardiovascular, renal and haemodynamic benefits, support the concept that SGLT2 inhibitors act as systemic modulators of metabolic and inflammatory stress, in both diabetic and non-diabetic populations.

Given that metabolic dysregulation, glucotoxicity, oxidative stress, and chronic low-grade inflammation represent central mechanisms not only in cardiometabolic disease but also in neurodegenerative disorders, these systemic effects provide a mechanistic foundation for exploring how metabolic stress and glucotoxicity may act as drivers of neurodegeneration.

### 2.2. Brain-Selective SGLT2 Inhibition: Safety Considerations in Elderly Patients

Although SGLT2 inhibitors have demonstrated significant cardiovascular and renal benefits in major randomised controlled trials [29,36], evidence regarding their tolerability in frail older adults with Alzheimer’s disease (AD) remains limited, as pivotal studies have largely excluded patients with cognitive impairment, frailty, and multimorbidity. At the same time, increasing preclinical evidence suggests that SGLT2 inhibitors may exert neuroprotective effects through modulation of neuroinflammation, mitochondrial function, oxidative stress, and cerebral insulin signalling pathways [42,43].

From a clinical perspective, the main issues regarding the tolerability of SGLT2 inhibitors in elderly patients relate less to severe hypoglycaemia and more to osmotic and haemodynamic effects. Although hypoglycaemic adverse events generally appear comparable to those of placebo when SGLT2 inhibitors are used without concomitant insulin or sulphonylureas, older adults may be more vulnerable due to polypharmacy, frailty, baseline hypotension and impaired volume regulation [44,45]. Based on various studies conducted on dapagliflozin, empagliflozin and canagliflozin, hypoglycaemia rates were generally low and similar to those of placebo, whilst volume depletion events were more frequent in patients aged ≥75 years, particularly in those receiving concomitant antihypertensive therapy or who had baseline hypotension [44,46,47].

Similarly, no significantly higher incidence of urinary and genital infections was observed compared with placebo, although numerically higher rates were reported in several studies, particularly among women [46,48].

Real-world data underscore the importance of renal monitoring in the elderly population. In a cohort study designed to assess changes in eGFR following the initiation of SGLT2 inhibitor therapy, approximately one-fifth of patients experienced an early decline in renal filtration, with the highest incidence observed in individuals aged 80 years or older. However, it is important to note that renal function subsequently stabilized over time across all age groups, supporting the interpretation of this phenomenon as a transient hemodynamic effect rather than progressive renal damage [49].

A retrospective pharmacovigilance study was conducted, based on the FDA’s global database, to analyse safety reports relating to the use of SGLT2 inhibitors in adults (<75 years) and the elderly (≥75 years). Safety reports were identified for 129,795 patients who received non-insulin antidiabetic drugs (NIADs), including 24,253 who were treated with SGLT2i (median age 60 [IQR: 51–68] years, 2339 [9.6%] aged ≥75 years). Compared to other NIADs, SGLT2i were significantly associated with amputations (adj. ROR = 355.1 [95% CI: 258.8–487.3] vs. adj. ROR = 250.2 [79.3–789.5]), Fournier’s gangrene (adj. ROR = 45.0 [34.5–58.8] vs. adj. ROR = 88.0 [27.0–286.6]), diabetic ketoacidosis (adj. ROR = 32.3 [30.0–34.8] vs. adj. ROR = 23.3 [19.2–28.3]), genitourinary infections (adj. ROR = 10.3 [9.4–11.2] vs. adj. ROR = 8.6 [7.2–10.3]), nocturia (adj. ROR = 5.5 [3.7–8.2] vs. adj. ROR = 6.7 [2.8–15.7]), dehydration (adj. ROR = 2.5 [2.3–2.8] vs. adj. ROR = 2.6 [2.1–3.3]), and fractures (adj. ROR = 1.7 [1.4–2.1] vs. adj. ROR = 1.5 [1.02–2.1]) in both adults and older adults, respectively. None of these safety signals was significantly greater in older adults (P-interaction threshold of 0.05). Acute kidney injury was associated with SGLT2i in adults (adj. ROR = 1.97 [1.85–2.09]) but not in older adults (adj. ROR = 0.71 [0.59–0.84]). Falls, hypotension, and syncope were not associated with SGLT2i among either adults or older adults [50].

In this context, pharmacological heterogeneity within the SGLT2 inhibitor class may become clinically relevant. SGLT2 inhibitors are lipophilic compounds capable of crossing the blood–brain barrier, achieving brain-to-serum AUC ratios ranging from 0.3 for canagliflozin and dapagliflozin to 0.5 for empagliflozin [8], resulting in a distinction with direct pharmacodynamic relevance, as SGLT1 receptors are widely expressed in hippocampal and cortical neurons implicated in the pathophysiology of AD [8,51].

The use of molecules with a higher brain-to-serum ratio and greater selectivity for SGLT2 could therefore, in theory, maximise interaction with central neuroprotective targets—including the upregulation of brain-derived neurotrophic factor (BDNF), inhibition of acetylcholinesterase activity and suppression of NLRP3-mediated neuroinflammation [8,52]. This approach would reduce the systemic osmotic load responsible for poor tolerability in frail elderly patients, which leads to volume depletion, orthostatic hypotension, urinary tract infections, and the transient decline in eGFR observed at the start of treatment [46,50,53]. This strategy of exploiting the differential binding profiles between the central nervous system and the kidneys to optimize the therapeutic index within the class of SGLT2 inhibitors represents a compelling but as yet unvalidated hypothesis, and prospective studies specifically designed for elderly populations with cognitive impairment are urgently needed to assess its clinical feasibility [54].

This strategy, which is based on exploiting the various modes of interaction between the central nervous system and the kidneys to optimize the therapeutic index of SGLT2 inhibitors, represents a promising hypothesis but has yet to be definitively confirmed. Prospective studies specifically designed for elderly populations with cognitive impairment are therefore needed to assess its actual clinical feasibility [54].

## 3. Metabolic Stress and Glucotoxicity as Drivers of Neurodegeneration: Implications for Alzheimer’s Disease

The systemic metabolic benefits observed with SGLT2i, including improved glucose homeostasis, attenuation of inflammatory signaling, and reduction of oxidative stress, highlight the central role of metabolic dysregulation in disease pathophysiology. While these effects have been primarily characterized in peripheral organs such as the kidney, heart, and vasculature, growing evidence indicates that chronic metabolic stress also profoundly affects the central nervous system. Sustained hyperglycemia and glucose-mediated toxicity, collectively referred to as glucotoxicity, have emerged as key contributors to neuronal dysfunction and neurodegeneration.

Glucotoxicity, defined as sustained cellular damage induced by prolonged exposure to elevated glucose concentrations, has emerged as a critical link between diabetes mellitus and cognitive decline, particularly in AD, the most common form of dementia. Under conditions of chronic hyperglycemia, excess intracellular glucose is diverted into several alternative metabolic pathways, including NADPH oxidase activation, the polyol pathway, the hexosamine biosynthetic pathway, and advanced glycation end-product (AGE) formation. These interconnected processes collectively amplify oxidative stress, inflammation, and neuronal dysfunction.

In the brain, chronic metabolic stress disrupts neuronal redox balance, insulin signaling, and mitochondrial function, ultimately driving synaptic failure and neuronal loss. These alterations closely mirror several molecular features observed in Alzheimer’s disease in both preclinical models and clinical investigations, including oxidative damage, impaired glucose metabolism, and chronic neuroinflammatory activation [55,56].

### 3.1. Oxidative Stress

Oxidative stress represents one of the earliest pathological events observed in Alzheimer’s disease and is strongly exacerbated under conditions of glucotoxicity. Indeed, chronic hyperglycemia promotes excessive production of ROS, leading to an imbalance between pro-oxidant and antioxidant systems and resulting in oxidative damage to lipids, proteins, and nucleic acids [57,58].

ROS include both radical species such as superoxide (O_2_^•−^), hydroxyl radicals (OH^•^), and nitric oxide (NO^•^), as well as non-radical oxidants including hydrogen peroxide (H_2_O_2_), which can propagate oxidative reactions within cells. Indeed, dysregulation of ROS homeostasis affects multiple cellular pathways and organelle functions, ultimately contributing to the onset and progression of a wide range of pathological conditions [59,60,61,62,63,64].

**Figure 1:**
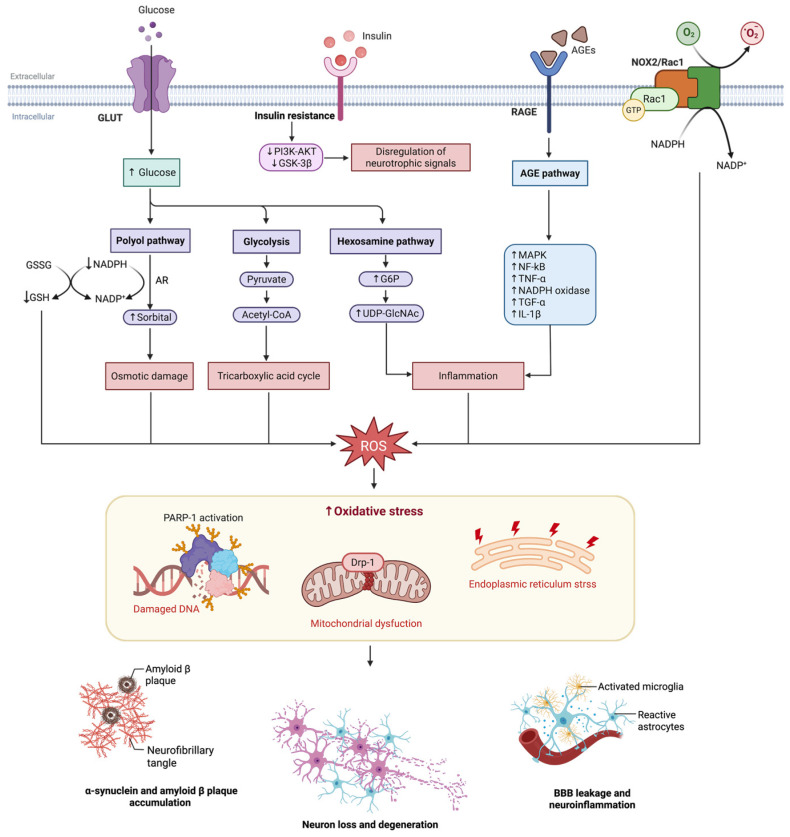
Molecular mechanisms of glucotoxicity-induced neurodegeneration. In conditions of chronic hyperglycemia, elevated intracellular glucose levels are diverted through the polyol pathway, where aldose reductase (AR) catalyzes the reduction of glucose to sorbitol, a process that depletes nicotinamide adenine dinucleotide phosphate (NADPH) pools and compromises the regeneration of reduced glutathione (GSH), thereby weakening cellular antioxidant defenses. Simultaneously, increased flux through the hexosamine biosynthetic pathway (HBP) leads to increased uridine diphosphate N-acetylglucosamine (UDP-GlcNAc) and aberrant protein O-linked β-N-acetylglucosamine (O-GlcNAc) modification (O-GlcNAcylation), disrupting protein homeostasis, while parallel increases in glycolysis and tricarboxylic acid (TCA) cycle activity further contribute to metabolic strain. On the neuronal membrane, the non-enzymatic formation and binding of advanced glycation end-products (AGEs) to the receptor for advanced glycation end-products (RAGE) initiates sustained nuclear factor kappa-light-chain-enhancer of activated B cells (NF-kB)-dependent inflammatory signaling and activates stress-responsive mitogen-activated protein kinases (MAPK), such as c-Jun N-terminal kinase (JNK) and p38. Concurrently, glucose-induced activation of the small GTPase Ras-related C3 botulinum toxin substrate 1 (Rac1) promotes the assembly of the NADPH oxidase 2 (NOX2) complex, generating a massive burst of reactive oxygen species (ROS). These events converge with brain insulin resistance, characterized by impaired phosphoinositide 3-kinase (PI3K) and protein kinase B (Akt) signaling, to activate glycogen synthase kinase 3 β (GSK3β), which promotes dynamin-related protein 1 (Drp1)-mediated mitochondrial fission and endoplasmic reticulum (ER) stress. In the nucleus, ROS-induced DNA damage overactivates poly(ADP-ribose) polymerase 1 (PARP-1), which exhausts cellular nicotinamide adenine dinucleotide (NAD^+^) and adenosine triphosphate (ATP) pools, leading to a total metabolic collapse. Collectively, these oxidative and inflammatory stresses drive the amyloidogenic processing of amyloid precursor protein (APP) into amyloid-β (Aβ) plaques and the hyperphosphorylation of Tau protein into neurofibrillary tangles, alongside microglial and astrocyte activation and blood–brain barrier (BBB) leakage, ultimately resulting in synaptic dysfunction and neuronal death. Created with BioRender.com.

In metabolic disorders such as type 2 diabetes, persistent hyperglycemia and insulin resistance markedly enhance ROS production and disrupt cellular redox homeostasis. Excess ROS activate stress-sensitive signaling pathways including NF-κB, JNK/SAPK (c-Jun N-terminal Kinase/Stress-Activated Protein Kinase), and p38 MAPK, which promote inflammatory responses, metabolic dysfunction, and cellular damage (Figure 1) [65]. At the same time, chronic metabolic stress impairs endogenous antioxidant defenses. Experimental diabetic models exhibit reduced activity of antioxidant enzymes such as catalase and superoxide dismutase (SOD) together with increased lipid peroxidation in brain regions critical for cognition, including the hippocampus and cortex [66,67].

These alterations destabilize physiological redox signaling and establish a self-reinforcing cycle in which oxidative stress further aggravates insulin resistance and metabolic dysfunction [65,68].

The brain is particularly susceptible to oxidative injury due to its high metabolic demand, elevated oxygen consumption, and abundance of polyunsaturated lipids that are highly vulnerable to peroxidation [69].

Consistent with this vulnerability, clinical and post-mortem studies have shown that oxidative damage is detectable during early and even preclinical stages of Alzheimer’s disease, as reflected by increased levels of oxidative biomarkers such as F2-isoprostanes, malondialdehyde, and 4-hydroxynonenal in brain tissue and biological fluids [70].

Importantly, oxidative stress not only reflects neuronal injury but also actively contributes to Alzheimer’s disease pathology. Excessive ROS production promotes amyloidogenic processing of amyloid precursor protein by increasing β-secretase and γ-secretase activity, thereby enhancing amyloid-β accumulation. In parallel, activation of stress-responsive kinases such as p38 MAPK and glycogen synthase kinase-3β (GSK-3β) facilitates tau hyperphosphorylation and neurofibrillary tangle formation [68,71,72].

Under glucotoxic conditions, sustained ROS production amplifies inflammatory signaling, disrupts neuronal metabolic homeostasis, and promotes amyloid and tau pathology, thereby contributing to synaptic dysfunction and cognitive decline.

#### 3.1.1. Mitochondrial ROS Production

Evidence from experimental diabetic and neurodegenerative preclinical models strongly supports the role for mitochondrial dysfunction in glucotoxic neuronal injury. Mitochondrial dysfunction represents an important mechanistic link between glucotoxicity, oxidative stress, and neuronal degeneration in metabolic disorders and Alzheimer’s disease. Mitochondria are highly dynamic organelles that constantly undergo cycles of fission and fusion, processes that are essential for maintaining mitochondrial integrity, bioenergetic efficiency, and cellular homeostasis [73,74,75].

Mitochondrial fission is mainly regulated by dynamin-related protein-1 (Drp1), whereas mitochondrial fusion is controlled by the large dynamin-related GTPases mitofusin-1 (Mfn1), mitofusin-2 (Mfn2), and optic atrophy-1 (OPA1). The balance between these processes determines mitochondrial morphology, respiratory enzyme activity, and ATP production [76].

Chronic hyperglycemia alters mitochondrial dynamics and favors excessive mitochondrial fission. In diabetic conditions, this imbalance is largely associated with increased Drp1 expression, which induces mitochondrial fragmentation, impaired oxidative phosphorylation, and reduced ATP generation in several cell types, including pancreatic β-cells and dorsal root ganglion neurons (Figure 1) [77,78].

These alterations contribute to the development of insulin resistance and persistent hyperglycemia, highlighting the role of mitochondrial bioenergetic impairment in metabolic disease [79].

Experimental studies further demonstrate that mitochondrial dysfunction contributes directly to neuronal and synaptic impairment in diabetes. In db/db mice, a widely used model of type 2 diabetes, increased Drp1 expression and enhanced mitochondrial fission have been observed in dorsal root ganglion neurons as well as in hippocampal neurons. These changes are accompanied by reduced mitochondrial respiratory activity, including decreases in complex I activity, together with altered ATP levels in hippocampal tissue. Pharmacological or genetic inhibition of Drp1 prevents mitochondrial fragmentation and restores mitochondrial function in neurons exposed to hyperglycemic conditions, indicating a central role of altered mitochondrial dynamics in glucotoxic neuronal damage. Upstream regulatory mechanisms also contribute to this process. Glycogen synthase kinase-3β (GSK3β) has been identified as a key signaling regulator of Drp1-mediated mitochondrial fission. Activation of GSK3β promotes Drp1 upregulation and mitochondrial fragmentation, whereas inhibition of GSK3β prevents hyperglycemia-induced mitochondrial dysfunction. The GSK3β–Drp1 signaling axis has also been associated with synaptic impairment, including reduced long-term potentiation, linking mitochondrial dysfunction to cognitive deficits observed in diabetic neuropathy and metabolic brain injury. Therefore, the glucotoxicity disrupts mitochondrial dynamics and bioenergetic function through mechanisms involving Drp1-dependent mitochondrial fission and GSK3β signaling, leading to impaired ATP production, enhanced oxidative stress, and synaptic dysfunction [80,81].

Mitochondrial alterations represent a critical mechanistic bridge between metabolic stress and neurodegenerative processes associated with chronic hyperglycemia.

#### 3.1.2. NADPH Oxidase Activation

Elevated glucose levels promote overload of the mitochondrial electron transport chain and activate NADPH oxidases (NOX), particularly NOX2, through a Rac1 GTPase (Ras-related C3 botulinum toxin substrate 1)-dependent assembly mechanism. This process enhances the generation of superoxide anions and amplifies cellular oxidative damage. Although this mechanism was initially described in endothelial and cardiac cells, it has subsequently been confirmed in neurons and microglia, where NOX-derived ROS contribute to synaptic dysfunction, neuroinflammation, and the progression of neurodegeneration (Figure 1) [82,83].

Rac1 plays a critical role in NOX2 NADPH oxidase activation by facilitating the recruitment of the cytosolic regulatory subunits, such as p67phox, a key component of the NOX2 complex, to the membrane, thereby promoting assembly of the active enzymatic complex and increasing ROS production. Preclinical studies have shown that pharmacological inhibition of NADPH oxidase or its upstream activator Rac1 significantly attenuates oxidative stress–mediated neuronal damage and improves cognitive performance in relevant experimental models of cerebral ischemia, diabetes, and neurodegeneration. For example, inhibition of Rac1 using NSC23766 reduced NADPH oxidase activation, ROS production, and neuronal cell death in rodent models of cerebral ischemia, preserving hippocampal integrity and cognitive function. Similarly, suppression of NOX activity has been shown to reduce diabetes-induced ROS generation and restore cognitive function in streptozotocin-induced diabetic rats, an effect also observed in animals treated with empagliflozin [84,85].

Collectively, these preclinical findings indicate that the Rac1/NOX2/ROS axis represents a relevant mechanistic link between glucotoxicity, oxidative stress, and neuronal damage, ultimately contributing to synaptic dysfunction and cognitive decline in neurodegenerative conditions associated with metabolic dysregulation.

#### 3.1.3. DNA Damage and PARP-1 Activation

Oxidative stress-induced DNA damage is another mechanism linking metabolic dysregulation to neuronal injury in AD. Sustained ROS production under chronic hyperglycemia results in extensive oxidative DNA damage, a potent activator of poly (ADP-ribose) polymerase-1 (PARP-1). Although PARP-1 normally participates in DNA repair, excessive activation leads to the formation of poly (ADP-ribose) polymers at the expense of intracellular nicotinamide adenine dinucleotide (NAD^+^), depleting NAD^+^ pools and consequently reducing ATP production (Figure 1).

This metabolic collapse impairs cellular energy homeostasis and promotes neuronal dysfunction and death. In preclinical AD and other neurodegenerative models, PARP-1 overactivation has been associated with mitochondrial dysfunction, bioenergetic failure, and activation of cell death pathways, including parthanatos [86,87].

Genetic ablation or pharmacological inhibition of PARP-1 reduces oxidative damage, mitigates amyloid pathology and neuroinflammation in AD models, and improves synaptic integrity and cognitive performance [88].

In peripheral and central diabetic models, PARP activation correlates with oxidative-nitrosative stress and metabolic dysfunction. In streptozotocin-induced diabetic rats, PARP inhibition reduced oxidative stress markers in peripheral nerve and spinal cord and improved nerve function, supporting its role in hyperglycemia-linked neuronal and glial dysfunction [89].

#### 3.1.4. Polyol Pathway Activation and Redox Imbalance

Although traditionally associated with diabetic complications, activation of the polyol pathway also contributes to oxidative stress mechanisms relevant to Alzheimer’s disease. Sustained hyperglycemia promotes a maladaptive redistribution of glucose flux toward the polyol pathway, a normally minor metabolic route that becomes highly active under glucotoxic conditions. In this pathway, aldose reductase (AR) catalyzes the reduction of glucose to sorbitol using NADPH as a cofactor, thereby competing with antioxidant systems for NADPH availability. Chronic activation of this reaction leads to NADPH depletion, impaired regeneration of reduced glutathione (GSH), and progressive weakening of cellular antioxidant defenses (Figure 1). Therefore, neurons exposed to high glucose become particularly vulnerable to oxidative stress and mitochondrial dysfunction, two central features of neurodegenerative processes.

Experimental evidence supports a direct role of polyol pathway activation in glucotoxic neuronal injury. In neuronal and neural progenitor cell cultures, high-glucose exposure increases AR expression, ROS accumulation, and apoptotic cell death, while pharmacological inhibition of AR significantly attenuates oxidative stress and preserves cell viability. These findings indicate that excessive polyol pathway flux is sufficient to induce neuronal damage independently of other diabetic complications, acting as a primary driver of glucotoxicity at the cellular level [90,91].

Beyond peripheral neuropathy, increasing evidence suggests that aldose reductase–dependent redox imbalance may also contribute to central neurodegenerative mechanisms. Aldose reductase is expressed in the brain and has been implicated in neuroinflammatory signaling and oxidative stress responses relevant to Alzheimer’s disease. In microglial models, β-amyloid stimulation enhances ROS production and inflammatory mediator release through AR-dependent mechanisms, while AR inhibition reduces oxidative stress and limits neurotoxic inflammatory signaling toward neighboring neurons [92].

### 3.2. Hexosamine Biosynthetic Pathway and O-GlcNAc Dysregulation

Alterations in glucose metabolism also influence protein modification pathways implicated in AD pathology. In parallel with other glucose-driven metabolic stress pathways, elevated intracellular glucose increases flux through the hexosamine biosynthetic pathway (HBP), leading to altered protein O-linked β-N-acetylglucosamine (O-GlcNAc) modification. The HBP utilizes a small fraction of cellular fructose-6-phosphate to generate UDP-GlcNAc, the donor substrate for protein O-GlcNAcylation (Figure 1). Under acute stress, increases in O-GlcNAcylation can function as an adaptive response that supports protein homeostasis and cell survival by modulating key signaling and stress pathways. However, chronic perturbations in HBP flux and persistent O-GlcNAcylation are associated with maladaptive changes that compromise neuronal function and viability. Disruption of O-GlcNAc cycling has been proposed to contribute to several neurodegenerative processes, linking aberrant glucose metabolism to neuronal dysfunction and disease progression [93].

Impaired glucose metabolism, a hallmark of both diabetes and AD, is associated with dysregulated protein O-GlcNAcylation in the brain. Clinical and post-mortem studies of human AD and diabetic brain tissues show decreased global O-GlcNAcylation and disrupted O-GlcNAc regulation, which correlate with increased pathological tau phosphorylation and cognitive impairment. Specifically, reduced O-GlcNAcylation of tau is inversely associated with its abnormal hyperphosphorylation, a key driver of neurofibrillary degeneration in AD pathology. Complementary evidence from preclinical models further supports this relationship. Experimental inhibition of the HBP in rat brain also reduces O-GlcNAc levels and increases tau phosphorylation, recapitulating features of impaired glucose metabolism and reinforcing the connection between HBP dysfunction and tau pathology [94].

Preclinical models further support the contribution of altered O-GlcNAc signaling to metabolic stress-driven neurodegeneration. In aging mouse models with metabolic syndrome, reduced O-GlcNAc signaling accompanies hyperphosphorylated tau accumulation and cognitive deficits, suggesting a mechanistic link between systemic metabolic dysfunction, HBP disruption, and neurodegenerative phenotypes [95].

Additionally, studies in peripheral neurons indicate that high-glucose conditions can increase O-GlcNAcylation, leading to aberrant neuronal activity and cellular stress, which can be ameliorated by pharmacological inhibition of O-GlcNAc cycling [96].

### 3.3. AGE-RAGE Signaling and Inflammatory Cascades

The progressive accumulation of advanced glycation end-products (AGEs) represents a major mechanism linking glucotoxicity to Alzheimer’s disease pathology. AGEs are generated through non-enzymatic glycation and oxidation reactions that occur under conditions of chronic hyperglycemia.

In the central nervous system, AGEs accumulate in neurons, astrocytes, and cerebral microvessels, where they exert deleterious effects primarily through activation of the receptor for AGEs (RAGE). Engagement of AGE–RAGE initiates sustained NF-κB–dependent inflammatory signaling and activates MAPK pathways, including JNK and p38, thereby promoting oxidative stress, synaptic dysfunction, and pro-apoptotic cascades (Figure 1). In vitro studies in neuronal cultures demonstrate that AGE exposure potentiates amyloid-β (Aβ) induced toxicity, enhances oxidative damage, and accelerates synaptic loss through RAGE-dependent mechanisms [97,98].

In vivo, multiple diabetic and AD murine models consistently demonstrate increased cerebral accumulation of AGEs and upregulation of their receptor RAGE, which correlate with synaptic degeneration and cognitive impairment. For example, in long-term diabetic mice (18–33 weeks of sustained hyperglycemia), elevated RAGE expression in neurons and glial cells was associated with impaired spatial memory performance and increased hippocampal Aβ and RAGE immunoreactivity, suggesting a direct link between hyperglycemia-driven AGE–RAGE activation and cognitive dysfunction [99]. In genetic AD models, mice overexpressing both mutant amyloid precursor protein (mAPP) and RAGE exhibit exacerbated learning and memory deficits and accelerated neuropathological changes compared with mAPP controls; in contrast, mice expressing a dominant-negative form of RAGE (DN-RAGE) maintain better spatial learning and reduced pathology, indicating that RAGE expression levels modulate disease severity in vivo [100]. Additional studies manipulating RAGE in AD transgenics have shown that global RAGE deletion (mAPP/RO mice) reduces β- and γ-secretase activity, lowers cerebral Aβ accumulation, and attenuates learning and memory impairments, further supporting the causal role of RAGE in amyloid pathology and cognitive decline [101]. Collectively, these preclinical findings indicate that AGE–RAGE signaling directly contributes to synaptic dysfunction and cognitive decline associated with metabolic stress. Genetic deletion or pharmacological inhibition of RAGE has been shown to attenuate neuroinflammation, reduce neuronal damage, and improve cognitive performance across different models, underscoring the pathogenic relevance of this axis. In AD mouse models, targeted disruption of RAGE signaling, either through dominant-negative constructs or conditional RAGE knockout in neurons, prevents AGE-induced synaptic dysfunction, preserves hippocampal long-term potentiation (LTP), and blocks p38 MAPK activation, which is a key downstream effector of RAGE-mediated inflammatory signaling [102]. In diabetic db/db mice, treatment with the RAGE inhibitor FPS-ZM1 significantly reduces pathological Aβ influx across the blood–brain barrier, decreases NF-κB signaling and apoptosis, and restores hippocampal plasticity and cognitive performance in behavioral tests such as the Morris water maze, without affecting systemic glucose levels, demonstrating that RAGE blockade can directly ameliorate glucotoxic brain injury [103]. Additionally, emerging work in diabetic mice has identified novel agents targeting the interaction between RAGE and intracellular mediators such as RIPK1; these compounds disrupt RAGE-associated inflammatory complexes in microglia, decrease neuroinflammation and dendritic damage, and prevent diabetes-associated cognitive deficits, suggesting innovative avenues for therapy beyond traditional receptor antagonism [104].

### 3.4. Brain Insulin Resistance and Impaired Neuronal Glucose Metabolism

A key intersection between glucotoxicity and neurodegeneration lies in brain insulin resistance, a condition increasingly associated with Alzheimer’s disease and sometimes described as “type 3 diabetes” [4]. This concept is supported by both clinical observations in AD patients and mechanistic evidence derived from experimental models. Insulin receptors are widely expressed in hippocampal and cortical neurons, where insulin signaling modulates synaptic plasticity, neuronal survival, and glucose utilization. Alterations in neuronal insulin signaling are evident in AD and in metabolic disease, contributing to cognitive decline and synaptic dysfunction.

Under physiological conditions, insulin stimulates the translocation of the insulin-sensitive glucose transporter GLUT4 to the neuronal plasma membrane. Preclinical studies demonstrated that intracerebroventricular insulin increases phosphorylation of Akt and drives GLUT4 insertion into the hippocampal neuron membrane in a PI3K–dependent manner, suggesting a mechanism by which neuronal insulin signaling may acutely enhance glucose uptake in active circuits. In neuronal cell models, both insulin and leptin induce GLUT4 translocation and increase glucose uptake via PI3K-dependent signaling, while chronic exposure to insulin downregulates GLUT4 and impairs acute glucose uptake responses, paralleling features of insulin resistance in the brain [105]. Consistent with these mechanistic insights, hippocampal GLUT4 is dynamically mobilized during memory encoding: contextual learning increases GLUT4 association with the plasma membrane in rats, and selective blockade of GLUT4-mediated glucose transport impairs insulin-enhanced spatial working memory and reduces neuronal glucose utilization in vitro and in vivo [106]. These findings support a model in which functional GLUT4 is required for the pro-cognitive effects of elevated insulin signaling in the hippocampus and suggest that impaired GLUT4 trafficking contributes to cognitive deficits when insulin signaling is disrupted. In pathological states of chronic hyperglycemia and elevated Aβ, insulin signaling in the brain is perturbed. Aβ oligomers impair insulin receptor autophosphorylation, inhibit Akt activation, and reduce GLUT4 membrane localization in hippocampal neurons, leading to diminished glucose uptake, hypometabolism, and synaptic dysfunction [107]. Consistent with these mechanistic findings, preclinical studies in AD mouse models and in diet-induced insulin-resistant rodents, reduced neuronal insulin signaling correlates with decreased GLUT4 activation, synaptic loss, and memory impairment. Restoration of insulin signaling via intranasal insulin treatment or insulin sensitizers, improves hippocampal plasticity and cognitive performance, further establishing brain insulin resistance as a driver of neurodegeneration [108].

## 4. Evidence for Neuroprotective Effects of SGLT2 Inhibitors

SGLT2 inhibitors (SGLT2i) exert multiple pleiotropic effects that extend beyond their primary role in glucose lowering. Their neuroprotective effects appear to arise from a combination of systemic metabolic improvements and direct actions within the central nervous system, including modulation of oxidative stress, neuroinflammation, mitochondrial function, and cerebrovascular integrity [8,109,110].

Experimental studies indicate that SGLT2 inhibition can attenuate neuronal injury under metabolic and ischemic stress. In vitro oxygen–glucose deprivation (OGD) models demonstrate that empagliflozin and dapagliflozin reduce apoptotic signaling and preserve neurovascular unit integrity [111]. Consistent with these observations, middle cerebral artery occlusion models show that SGLT2 inhibition reduces infarct volume and improves neurological outcomes. In hyperglycemic rodents, empagliflozin attenuates ischemia–reperfusion injury by limiting oxidative stress, suppressing inflammatory cytokine production, and stabilizing BBB integrity [112]. Comparable neuroprotective effects have also been reported in traumatic brain injury models, where SGLT2 inhibition reduces cerebral edema, lesion size, and neurological deficits [113,114].

Evidence from neurodegenerative models further supports the capacity of SGLT2 inhibitors to influence mitochondrial and oxidative stress pathways. In rotenone-induced models of Parkinson’s disease, dapagliflozin improves motor performance and reduces lipid peroxidation, indicating attenuation of oxidative damage in dopaminergic neurons [115]. Mechanistically, these effects involve activation of the PI3K/Akt/GSK-3β pathway and increased expression of glial cell-derived neurotrophic factor, mechanisms that promote mitochondrial stability and reduce neuronal apoptosis [116].

In models of metabolic brain injury, SGLT2 inhibition also improves cognitive and vascular outcomes. In high-fat diet and streptozotocin-induced diabetic rodents, SGLT2 inhibitors restore tight junction protein expression, including ZO-1 and occludin, thereby preventing BBB disruption and the development of a “leaky brain” phenotype [117]. Additional studies report reductions in oxidative stress and inflammatory signaling pathways in metabolic neurodegeneration models treated with empagliflozin or dapagliflozin [118,119].

Translational evidence in humans is increasingly consistent with these experimental observations. Large population-based cohort studies report that treatment with SGLT2 inhibitors is associated with a significantly lower incidence of AD and vascular dementia compared with other antidiabetic therapies [120,121,122].

Meta-analyses similarly estimate an approximately 30–35% reduction in dementia risk among patients receiving SGLT2 inhibitors [123,124,125]. Mechanistic reviews suggest that these clinical associations may reflect the combined metabolic, vascular, and anti-inflammatory effects of SGLT2 inhibition [126,127].

Evidence for potential direct CNS involvement is supported by the detection of SGLT1 and SGLT2 expression in hippocampal regions involved in learning and memory, including the CA1 and CA3 areas and the dentate gyrus [113,128].

Increased expression of these transporters has also been reported in post-mortem brain tissue following traumatic brain injury [114,115].

In addition, SGLT2 expression in choroid plexus epithelial and ependymal cells suggests a potential role in cerebrospinal fluid homeostasis and brain metabolic regulation [129,130,131]. Through these combined systemic and central mechanisms, SGLT2 inhibition may influence several of the molecular pathways previously discussed in Alzheimer’s disease, including oxidative stress, mitochondrial dysfunction, neuroinflammation, and cerebrovascular damage.

### CNS Target Engagement: SGLT2 Selectivity and SGLT1 Off-Targeting

A fundamental question in evaluating the neuroprotective potential of gliflozins is whether their benefits arise from systemic metabolic correction or direct CNS engagement. SGLT2i are lipophilic agents capable of crossing the BBB, achieving brain-to-serum AUC ratios ranging from 0.3 for canagliflozin and dapagliflozin to 0.5 for empagliflozin [132]. However, SGLT2 expression in the human brain is extremely low and restricted mainly to microvessels, which contrasts with the robust neuroprotection observed in preclinical models [133].

Emerging evidence suggests that these cognitive benefits may significantly reflect SGLT1 off-targeting. Unlike SGLT2, SGLT1 is abundantly expressed in the hippocampus, cerebral cortex, and BBB endothelial cells, areas critical for learning and memory [134]. SGLT1 plays a pivotal role in neuronal glucose sensing and sodium-coupled transport; its dysregulation is closely linked to impaired synaptic plasticity and neuroinflammation [135].

Recent comparative pharmacological analyses highlight that while empagliflozin and dapagliflozin are highly SGLT2-selective, canagliflozin and the dual inhibitor sotagliflozin exhibit potent SGLT1 inhibition at clinically relevant concentrations. In neuronal models, SGLT1 inhibition was shown to modulate glucose-sensing pathways and reduce sodium-coupled metabolic strain, suggesting that dual SGLT1/2 inhibition may provide a more robust defense against glucotoxicity-induced neuronal apoptosis than selective SGLT2 blockade [136]. Using in vitro models of the neurovascular unit, research has demonstrated that SGLT1 is a key regulator of glucose transport across the BBB. In these studies, pharmacological inhibition of SGLT1 prevented endothelial dysfunction and tight-junction degradation (ZO-1 and occludin) induced by high-glucose conditions, a mechanism that potentially limits the “leaky brain” phenotype in the early stages of AD [134]. In rodent models of metabolic syndrome and hypertension, SGLT1 upregulation in the hippocampus was found to correlate with impaired long-term potentiation (LTP) and increased neuroinflammation. Treatment with non-selective SGLT inhibitors or dual agents like sotagliflozin restored synaptic markers and reduced microglial activation (Iba-1 levels), indicating that CNS-localized SGLT1 might be a superior target for preserving cognitive reserve in patients with high metabolic risk [135].

The pharmacological profile of the gliflozin class therefore varies in its therapeutic index for the CNS. The dual inhibitory action of agents like canagliflozin or sotagliflozin may offer a synergistic advantage in AD by simultaneously improving systemic insulin sensitivity via SGLT2 and directly modulating neuronal bioenergetics and redox balance via SGLT1. Additional evidence suggests that canagliflozin may exert direct CNS effects beyond SGLT modulation through acetylcholinesterase (AChE) inhibition. Preclinical pharmacological analyses demonstrated that canagliflozin directly binds AChE at brain-relevant concentrations, with significant enzyme inhibition observed at approximately 3 μM following serum exposure to 10 μM concentrations. This interaction was associated with improved memory performance in experimental models, suggesting that certain gliflozins may combine metabolic and cholinergic neuroprotective properties. These findings further support the concept that structural optimization of brain-penetrant SGLT inhibitors could enhance CNS-specific target engagement and potentially amplify therapeutic efficacy in AD. Consequently, the heterogeneity in cognitive outcomes reported in clinical meta-analyses might be partially explained by the varying degrees of SGLT1 affinity and subsequent target engagement across different SGLT2i [135,136,137,138].

## 5. SGLT2 Inhibitors in Alzheimer’s Disease: Biochemical Insights

### 5.1. Metabolic Dysfunction and Insulin Resistance in Alzheimer’s Disease

Alzheimer’s disease is a progressive neurodegenerative disorder characterized by extracellular Aβ plaques, intracellular neurofibrillary tangles composed of hyperphosphorylated tau, synaptic dysfunction, and progressive cognitive decline. Beyond their systemic glycemic effects, experimental evidence suggests that SGLT2i influence multiple pathways relevant to AD by acting as metabolic sensors. Experimental studies suggest that these agents activate AMP-activated protein kinase (AMPK) and subsequently inhibit mammalian Target of Rapamycin (mTOR) signaling. This shift promotes autophagy, facilitating the proteostatic clearance of amyloid-β and hyperphosphorylated tau. Furthermore, AMPK activation enhances mitochondrial biogenesis via PGC-1α and improves mitophagy, restoring bioenergetic efficiency and reducing reactive oxygen species (ROS) generation [139,140]. These mechanisms may counteract key aspects of the metabolic dysfunction associated with AD pathogenesis [141,142].

Chronic hyperglycemia promotes amyloidogenic processing of amyloid precursor protein (APP) and enhances Aβ production. In SH-SY5Y neuronal cells exposed to high glucose (25 mM), impaired APP degradation leads to increase Aβ accumulation [143]. Metabolic stress further exacerbates synaptic dysfunction through oxidative and nitrosative stress pathways. Combined exposure to Aβ oligomers and high glucose increases NMDA receptor-mediated Ca^2+^ influx and nitric oxide production, promoting aberrant S-nitrosylation of proteins such as insulin-degrading enzyme (IDE) and dynamin-related protein 1 (Drp1), thereby impairing Aβ clearance and mitochondrial dynamics [144].

**Figure 2:**
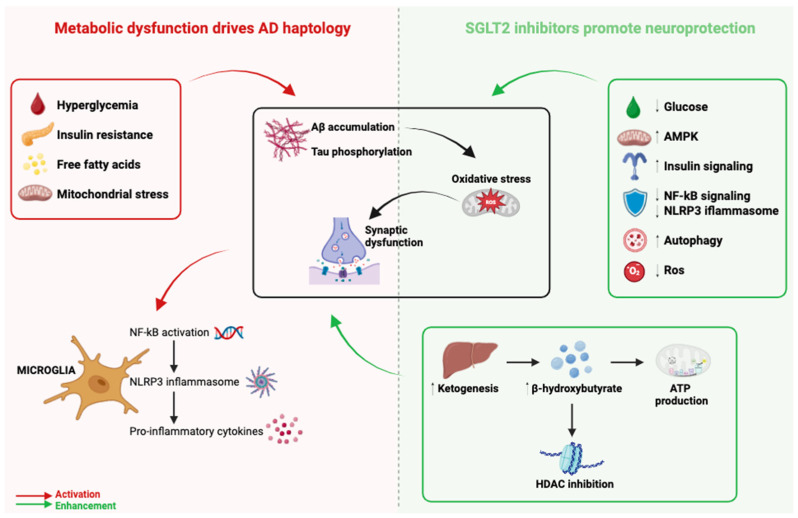
Schematic overview of metabolic dysfunction-driven neurodegeneration in Alzheimer’s disease (AD) and the protective effects of SGLT2 inhibitors. Hyperglycemia, insulin resistance, and mitochondrial stress promote Aβ accumulation, tau hyperphosphorylation, oxidative stress, synaptic dysfunction, and microglia-mediated neuroinflammation (NF-κB/NLRP3). SGLT2 inhibitors counteract these processes by improving glucose homeostasis, restoring insulin signaling, activating AMPK, reducing oxidative stress and inflammation, and enhancing autophagy. Additionally, increased ketogenesis leads to elevated β-hydroxybutyrate (BHB), which supports neuronal energy metabolism, modulates gene expression via HDAC inhibition, and attenuates neuroinflammation. Created with BioRender.com.

Impaired insulin signaling further exacerbates these defects by promoting tau hyperphosphorylation via PI3K/Akt dysregulation. SGLT2i counteract these processes; enavogliflozin improves cognitive function by attenuating neuroinflammation through suppression of NF-κB signaling and enhancement of microglial phagocytic activity via AMPK-mediated mitochondrial biogenesis [42,145]. Similarly, empagliflozin and dapagliflozin protect neurons from Aβ-induced toxicity by reducing ROS production, downregulating NLRP3 inflammasome activation, and attenuating tau pathology through modulation of key protein kinases [146]. Human genetic evidence from Mendelian randomization analyses further supports this association, indicating that SGLT2 inhibition is linked to a significantly lower likelihood of developing Alzheimer’s disease, with citrate proposed as a potential mediating metabolite [147]. Overall, the strongest evidence currently supports the systemic metabolic and anti-inflammatory effects of SGLT2i, whereas several CNS-specific neuroprotective mechanisms remain primarily supported by preclinical investigations. Together, these findings suggest that SGLT2 inhibitors may exert pleiotropic neuroprotective effects that extend beyond glucose lowering [148] (Figure 2).

### 5.2. Modulation of Neuroinflammation in AD

In parallel with metabolic remodeling, SGLT2 inhibitors attenuate neuroinflammatory signaling by suppressing NF-κB activation and NLRP3 inflammasome assembly, leading to decreased production of pro-inflammatory cytokines such as IL-1β and TNF-α. By modulating microglial polarization toward a less pro-inflammatory phenotype, these agents limit chronic synaptic injury. Additionally, improvements in endothelial nitric oxide bioavailability preserve the neurovascular unit and mitigate early BBB breakdown, which is increasingly recognized as a triggering event in AD [149,150,151].

The JAK2/STAT1 pathway plays a central role in this signaling; in experimental AD models, tau accumulation has been shown to activate JAK2, leading to phosphorylation of STAT1 and subsequent transcription of pro-inflammatory genes that impair synaptic function and neuronal survival. Persistent activation of this pathway contributes to a chronic inflammatory environment that accelerates neurodegeneration.

In 5XFAD mice, treatment with enavogliflozin significantly reduced Aβ plaque burden, suppressed NF-κB–mediated inflammatory responses, and improved cognitive performance, suggesting that SGLT2 inhibition attenuates inflammatory cascades associated with amyloid pathology [42].

Although primarily linked to NF-κB modulation, these findings support broader anti-inflammatory effects that may intersect with JAK/STAT signaling pathways.

In addition to regulating inflammatory transcriptional programs, SGLT2 inhibitors influence microglial activation states. Microglia can adopt a pro-inflammatory M1 phenotype, characterized by increased production of cytokines such as TNF-α, IL-1β, and IL-6, or an anti-inflammatory M2 phenotype that promotes tissue repair and Aβ clearance.

Experimental studies in T2D-AD murine models have shown that SGLT2 inhibition reduces microglial activation and promotes polarization toward the M2 phenotype, thereby enhancing phagocytic clearance of Aβ and reducing inflammatory cytokine production [152]. These immunomodulatory effects contribute to a reduction in neuroinflammatory signaling and improved neuronal homeostasis.

Mechanistic studies further indicate that SGLT2 inhibitors can modulate intracellular inflammatory signaling pathways directly. Empagliflozin has been shown to suppress activation of the JAK2/STAT1 axis in macrophages, reducing transcription of pro-inflammatory cytokines. Treatment of RAW 264.7 macrophages with increasing concentrations of empagliflozin significantly inhibited mRNA expression of inflammatory mediators, indicating a direct inhibitory effect on JAK2/STAT1-dependent transcriptional activity [153,154].

Beyond inflammatory modulation, in T2D–AD mouse, combining high-fat diet (HFD) feeding and streptozotocin administration demonstrate that SGLT2 inhibition (empagliflozin, 25 mg/kg/day) can restore impaired insulin signaling within the brain. In these models, SGLT2 inhibitor treatment improves peripheral insulin resistance while simultaneously restoring neuronal insulin signaling through activation of the IRS1/Akt/GSK3β pathway. Reactivation of this signaling cascade improves learning and memory behavior in T2D-AD mice and reduces pathological phosphorylation of tau protein. Mechanistically, inhibition of GSK3β, a key kinase involved in tau phosphorylation, appears to contribute to the reduction of hyperphosphorylated tau levels and stabilization of neuronal cytoskeletal dynamics [155].

Additional evidence indicates that SGLT2 inhibition may influence tau phosphorylation through interactions with the renin–angiotensin system (RAS). The RAS axis, traditionally associated with cardiovascular regulation, is increasingly recognized as an important modulator of brain metabolism and cognitive function. Angiotensin II signaling through the ACE/Ang II/AT1R axis promotes inflammation, insulin resistance, and oxidative stress, whereas activation of the ACE2/Ang(1-7)/Mas receptor pathway exerts neuroprotective effects. Experimental findings suggest that SGLT2 inhibitors may shift the balance toward the ACE2/Ang(1-7)/MasR axis, thereby improving neuronal insulin signaling and attenuating GSK3β-dependent tau phosphorylation. Through this mechanism, SGLT2 inhibition may reduce pathological accumulation of phosphorylated tau and mitigate neurodegenerative progression [155,156].

Finally, by improving glucose homeostasis, SGLT2i limit the formation of advanced glycation end products (AGEs) and their interaction with RAGE, thereby preventing NADPH oxidase-mediated ROS bursts and secondary BBB damage. By improving systemic glucose homeostasis and reducing chronic hyperglycemia, SGLT2 inhibition may also limit AGE formation, thereby decreasing RAGE-dependent amplification of oxidative injury.

Taken together, glucotoxicity contributes to Alzheimer’s disease progression through converging mechanisms involving amyloidogenesis, oxidative stress, insulin resistance, and chronic neuroinflammation. SGLT2 inhibitors counteract these processes at multiple molecular levels, suppressing NF-κB/NLRP3 inflammasome signaling, attenuating AGE–RAGE-mediated oxidative injury, modulating microglial activation, and restoring AMPK/mTOR-dependent autophagic pathways. These multimodal actions support the hypothesis that metabolic therapies targeting glucotoxicity may influence key biochemical drivers of neurodegeneration in Alzheimer’s disease (Figure 2).

### 5.3. Ketone Metabolism and Epigenetic Signaling

SGLT2i induce a metabolic state resembling fasting that promotes hepatic ketone body production. Inhibition of renal tubular glucose reabsorption leads to persistent glycosuria, resulting in reduced blood glucose and plasma insulin levels, accompanied by a relative increase in glucagon. This shift in the insulin-to-glucagon ratio promotes lipolysis in adipose tissue, increasing the release of non-esterified fatty acids (NEFAs), which reach the liver and are oxidized in mitochondria through β-oxidation. The increased mitochondrial production of acetyl-CoA not only provides energy for hepatic gluconeogenesis—by allosterically activating pyruvate carboxylase and facilitating the conversion of gluconeogenic substrates such as lactate and alanine—but, when the capacity of the tricarboxylic acid (TCA) cycle is exceeded, is also diverted toward ketogenesis, leading to the formation of acetoacetate and, in particular, β-hydroxybutyrate (BHB) [61]. The rise in circulating ketone bodies observed during SGLT2i therapy occurs rapidly and appears relatively independent of the improvement in insulin sensitivity associated with weight loss, suggesting a direct metabolic effect linked to the redistribution of energy substrates and possibly also to reduced renal clearance of ketones [157,158,159,160].

Within the central nervous system, BHB represents an alternative energy substrate that is particularly relevant in the context of AD, which is characterized by an early impairment of cerebral glucose metabolism. In this context, the mild ketosis induced by SGLT2 inhibitors may partially compensate for impaired cerebral glucose utilization associated with AD-related insulin resistance, thereby providing an alternative substrate to sustain neuronal mitochondrial ATP production and metabolic homeostasis.

After being transported across the BBB through monocarboxylate transporters (MCT1 and MCT2), BHB is converted to acetoacetate by mitochondrial β-hydroxybutyrate dehydrogenase and subsequently to acetyl-CoA via succinyl-CoA:3-oxoacid CoA transferase (SCOT), entering the TCA cycle and supporting ATP production. Beyond this bioenergetic role, BHB also acts as a signaling molecule capable of directly modulating epigenetic and inflammatory processes [161,162].

BHB has been shown to inhibit class I histone deacetylases (HDACs), including HDAC1, HDAC3 and HDAC4 [163].

Biochemical assays performed with purified enzymes suggest that this inhibition occurs through interaction of the BHB carboxylate group with the catalytic zinc ion located in the HDAC active site, a mechanism similar to that observed for other short-chain fatty acid HDAC inhibitors such as butyrate. These epigenetic effects have also been observed in vivo: conditions that elevate circulating BHB levels—such as fasting—increase histone acetylation in several murine tissues (C57BL/6 mice) [163,164], particularly at the promoter of the stress-responsive transcription factor Foxo3a, leading to increased Foxo3a expression through the relief of HDAC-mediated repression. Similarly, in the nervous system, exposure to BHB increases histone acetylation and promotes transcription of the brain-derived neurotrophic factor (Bdnf) gene in hippocampal neurons by reducing the occupancy of its promoter by HDAC2 and HDAC3 [165].

In parallel, BHB exerts relevant anti-inflammatory effects in the brain by inhibiting activation of the NLRP3 inflammasome in microglial cells, thereby reducing β-amyloid-induced inflammatory signaling and the production of pro-inflammatory cytokines such as IL-1β [166]. Overall, these findings suggest that the increase in ketone bodies induced by SGLT2i may contribute to neuroprotective effects through a combination of improved cerebral energy metabolism, epigenetic modulation, and attenuation of neuroinflammation [160]. This metabolic–signaling axis therefore represents a potential mechanism through which mild and controlled ketosis could influence the progression of neurodegenerative disorders, including Alzheimer’s disease (Figure 2).

While preclinical and observational data provide a compelling rationale for SGLT2i in AD, a critical distinction must be made between established systemic effects and hypothesis-generating CNS actions.

SGLT2i effectively reduce systemic glucotoxicity, improve vascular homeostasis, and modulate systemic inflammatory markers in both diabetic and non-diabetic populations. These systemic benefits are well-documented in large-scale clinical trials such as EMPA-REG OUTCOME [28,29] and DAPA-HF [38].

The direct modulation of microglial polarization toward an M2 phenotype and the specific activation of central AMPK/mTOR pathways largely rely on rodent models, such as the 5XFAD or db/db mice [42,152]. Translating these acute findings to the decade-long progression of human AD remains challenging. Furthermore, the discrepancy between positive real-world observational data [120,121,122] and neutral results in recent clinical meta-analyses [147,148] suggests that the neuroprotective efficacy of SGLT2i might be stage-specific or restricted to patients with high metabolic risk.

## 6. Clinical Studies, Translational Challenges, and Future Perspectives

Clinical evidence supporting the use of SGLT2 inhibitors in Alzheimer’s disease is still limited, but early findings are encouraging. In patients with type 2 diabetes, SGLT2i treatment not only enhances urinary glucose excretion but also improves β-cell insulin secretion and peripheral insulin sensitivity [167]. This is particularly relevant because cerebral glucose hypometabolism may precede the onset of AD symptoms by more than a decade [168].

Beyond preclinical findings, observational and emulated trial data suggest that SGLT2i therapy is associated with a reduced risk of AD and related dementias in patients with T2D compared with other glucose-lowering agents [169].

A recent comprehensive review further strengthened the mechanistic and translational rationale linking SGLT2 inhibition to reduced AD risk through anti-inflammatory, antioxidant, and metabolic effects [170].

Consistently, SGLT2i use has also been associated with a lower risk of dementia, Parkinson’s disease, and cerebrovascular mortality compared with DPP4 inhibitors in patients with T2D [171]. However, these observational risk reductions must be interpreted with significant caution. Such findings are often susceptible to confounding by indication, where the clinical profile of patients prescribed SGLT2 inhibitors fundamentally differs from those on older antidiabetic therapies. Furthermore, a healthy user bias may influence these cohorts, as patients receiving newer, pleiotropic drug classes often exhibit better health-seeking behaviors and treatment adherence. Additionally, unmeasured differences in diabetes severity and duration across treatment groups serve as critical confounders that may overstate the apparent neuroprotective efficacy of SGLT2 inhibitors. These methodological limitations likely contribute to the discrepancy between positive observational trends and the more neutral outcomes recently reported in large-scale meta-analyses of randomized controlled trials. In a 12-month randomized controlled study in older adults with T2D, SGLT2 inhibitors did not significantly improve cognitive performance compared with incretin-based therapy [172]. More recently, a pilot study repurposing dapagliflozin in patients with AD due to AD showed that the drug was generally well tolerated over 3 months, induced renal glucose disposal, and altered systemic metabolism, but did not significantly modify brain energy metabolism as assessed by MRS and FDG-PET. A modest improvement was observed only in Stroop Interference performance, while most other cognitive endpoints remained unchanged. Notably, dapagliflozin increased brain glutathione levels, suggesting a possible effect on cerebral redox balance, although this finding requires cautious interpretation [173].

The strongest evidence suggests that SGLT2 inhibitors have no effect, or at least no significant effect on Alzheimer’s disease and cognitive decline comes primarily from meta-analyses of randomised controlled trials, which temper the positive findings observed in observational studies. A recent meta-analysis conducted by Seminer et al., which examined 26 randomised clinical trials and 164,531 participants, found no significant association between cardioprotective hypoglycaemic therapies and a reduction in cognitive decline or dementia in general (odds ratio [OR] 0.83; 95% CI 0.60–1.14). However, subgroup analysis showed that GLP-1 receptor agonists (GLP-1RAs) were associated with a significant reduction in the risk of dementia (OR 0.55; 95% CI 0.35–0.86), whereas SGLT2 inhibitors were not (OR 1.20; 95% CI 0.67–2.17; P for heterogeneity = 0.04) [174]. Jaiswal et al. conducted a meta-analysis including 12 randomized controlled trials involving a total of 74,442 participants, of whom 40,784 received SGLT2 inhibitors and 33,658 were assigned to control groups. The mean age of participants was comparable between groups (65.3 years in the SGLT2i group vs. 65.2 years in controls). The pooled analysis demonstrated that the use of SGLT2 inhibitors was not significantly associated with a reduced risk of dementia overall (OR 1.37; 95% CI 0.70–2.69; *p* = 0.36), Alzheimer’s disease-type dementia (OR 1.99; 95% CI 0.59–6.71; *p* = 0.27), vascular dementia (OR 0.40; 95% CI 0.09–1.85; *p* = 0.24), or Parkinson’s disease (OR 0.63; 95% CI 0.25–1.61; *p* = 0.33) compared with control groups [123]. Similarly, a meta-analysis conducted by Xu et al., which examined 52 publications and 111,376 participants, assessed the neurological effects of SGLT2 inhibitors compared with placebo. Overall, SGLT2 inhibitors did not significantly affect the incidence of nervous system disorders (RR 1.29; 95% CI 0.78–2.12; *p* = 0.32). However, subgroup analysis suggested a modest protective effect against Parkinson’s disease in patients with severe heart failure [175]. Finally, Tseng et al. conducted a network meta-analysis (NMA) to assess the potential preventive effects of SGLT2 inhibitors on neurodegenerative diseases and to identify the most effective treatment strategies. The analysis included 22 randomised controlled trials involving 138,282 participants (mean age 64.8 years; 36.4% women). Only dapagliflozin demonstrated a significant preventive effect, particularly against Parkinson’s disease (odds ratio [OR] = 0.28; 95% confidence interval [CI], 0.09–0.93) compared with controls. In contrast, neither GLP-1 receptor agonists nor other SGLT2 inhibitors showed significant preventive benefits for the neurodegenerative conditions examined, including Alzheimer’s disease [176].

Several translational challenges remain. First, most mechanistic and efficacy data derive from preclinical models, whereas human studies are still few, small, and short in duration. Second, the central effects of SGLT2 inhibitors may be limited by the relatively low expression of SGLT2 in the brain, although SGLT transporters are present in regions involved in cognition and energy homeostasis, including the hippocampus, choroid plexus, and blood–brain barrier endothelium [140,177,178].

Their lipophilicity allows some degree of brain penetration, with brain-to-blood ratios reported for dapagliflozin and empagliflozin [179], but it remains unclear whether this is sufficient to produce robust direct effects on neuronal bioenergetics. Third, although SGLT2 inhibitors may promote ketogenesis and potentially support alternative brain energy metabolism, this effect appears less pronounced in non-diabetic individuals and may not be sufficient on its own to induce measurable cerebral metabolic changes, as observed in the dapagliflozin pilot study.

Additional complexity comes from the possibility that some of the benefits of SGLT2 inhibitors may be mediated indirectly through systemic metabolic remodeling rather than direct CNS actions. For example, empagliflozin has been shown to increase plasma citrulline levels in patients with T2D [180], a finding of potential interest because reduced arginine and citrulline concentrations have been reported in AD, and citrulline supplementation improved spatial memory and reduced APP-related pathology in experimental models [181,182].

However, the relevance of this pathway remains uncertain, particularly because arginine-related findings in AD are inconsistent, and nitric oxide signaling may exert both protective and detrimental effects depending on disease stage and NOS isoform involvement [183,184,185].

A noteworthy barrier to the broader clinical application of SGLT2 inhibitors is their contraindication in type 1 diabetes (T1D), where they increase the risk of diabetic ketoacidosis (DKA), including euglycemic presentations. Adjunctive trials in T1D (EASE programme) recorded DKA rates of 3.3–4.3% versus 1.2% with placebo, and pharmacovigilance data suggest a sevenfold increase in DKA risk with SGLT2 inhibitor use [186]. This safety concern is particularly relevant given that T1D patients share several metabolic and inflammatory vulnerabilities that theoretically make them candidates for the neuroprotective benefits of SGLT2 inhibition. Emerging continuous ketone monitoring (CKM) technology may offer a path forward: by enabling real-time detection of rising β-hydroxybutyrate levels, CKM could allow early intervention before ketosis progresses to DKA, potentially expanding the safe use of SGLT2 inhibitors in T1D populations [187]. Prospective trials integrating CKM with SGLT2 inhibition in T1D are needed to test this hypothesis and define safe operational boundaries.

Future studies should therefore focus on longer randomized clinical trials in biomarker-defined AD populations, improved measures of brain target engagement, and careful monitoring of body composition, frailty, and renal safety. Although SGLT2 inhibitors are already FDA-approved for T2D and heart failure, their long-term safety in cognitively impaired and often frail older adults still requires further evaluation [160].

To maximize the therapeutic index of SGLT2i in neurodegenerative disorders, future research must prioritize pharmacological optimizations for enhanced CNS target engagement. Current gliflozins exhibit moderate BBB permeability, but medicinal chemistry efforts could focus on lipophilic modifications of the glycosyl or aglycone moieties to improve passive diffusion across the neurovascular unit [188,189].

Furthermore, recent medicinal chemistry studies have explored multiple structural modifications of gliflozin-based SGLT2i to expand their biological activity as well as the pharmacokinetic properties. For instance, scaffold modifications of classical C-glucoside inhibitors in thioglucosides or isocoumarine analogues have generated novel derivatives with preserved or improved biological activity and expanded target profiles [190,191,192].

Beyond chemical modification, advanced delivery systems represent a promising frontier. Nanotechnology-based strategies, such as lipid-based nanoparticles or polymeric nanocarriers, could be evaluated to achieve targeted brain delivery, bypassing systemic metabolic degradation and reducing peripheral side effects like euglycemic DKA [193,194]. Furthermore, the development of dual SGLT1/2 inhibitors, or CNS-specific SGLT1 antagonists, may offer superior efficacy in Alzheimer’s disease. Given that SGLT1 is more abundantly expressed in cortical and hippocampal neurons than SGLT2, agents like sotagliflozin—which already demonstrate systemic safety—could be further optimized for higher brain-to-serum exposure to directly modulate neuronal glucose sensing and synaptic integrity [195,196]. Finally, emerging evidence using murine models suggests that direct CNS activation via SGLT2 inhibition can also regulate autonomic cardiovascular activity, underscoring the broad physiological impact of central gliflozin action and the need for organ-targeted drug design [197,198].

Future studies should also explore combination strategies targeting complementary metabolic and neuroinflammatory pathways. In this context, combining SGLT2 inhibitors with intranasal insulin or glucagon-like peptide-1 receptor agonists (GLP-1RAs) may represent a promising therapeutic approach, as these agents modulate partially overlapping but complementary mechanisms involved in insulin signaling, neuroinflammation, mitochondrial function, and cerebral energy metabolism. Notably, preliminary translational studies combining intranasal insulin with semaglutide in older adults with metabolic dysfunction and cognitive impairment have suggested the feasibility of multimodal metabolic interventions targeting both peripheral and central insulin resistance. In parallel, recent reviews discussing GLP-1RAs and SGLT2 inhibitors in AD highlight the potential advantage of simultaneously targeting glucotoxicity, impaired neuronal bioenergetics, and chronic neuroinflammation through combination-based therapeutic strategies [142,199,200].

Overall, current evidence supports continued investigation of SGLT2 inhibitors as repurposable agents in AD, but their clinical utility will depend on clarifying whether their benefits arise predominantly from peripheral metabolic correction, direct central actions, or synergy with other interventions such as exercise or ketogenic strategies.

## 7. Conclusions

Several compounds originally developed to counteract metabolic stress in diabetes have shown protective effects against glucotoxicity in neural systems. Similarly, a broad range of molecules targeting the downstream effects of glucose-induced stress consistently attenuate neuronal damage. These findings demonstrate that glucotoxicity is not merely a metabolic by-product of diabetes but an active driver of neurodegenerative processes.

SGLT2 inhibitors have emerged as promising candidates in the context of Alzheimer’s disease due to their ability to modulate several biological processes implicated in neurodegeneration, including glucotoxicity, insulin resistance, oxidative stress, neuroinflammation, and impaired cellular energy metabolism. Extensive preclinical evidence indicates that these drugs can reduce amyloid- and tau-related pathology, improve neuronal homeostasis, and preserve cognitive function through interconnected metabolic and inflammatory pathways.

Importantly, the potential relevance of SGLT2 inhibitors in Alzheimer’s disease likely derives not from a disease-specific anti-Alzheimer mechanism, but from their ability to modulate systemic metabolic and inflammatory hubs that intersect with neurodegenerative processes. By improving peripheral metabolic homeostasis and influencing signaling pathways involved in energy balance, inflammation, and cellular stress responses, SGLT2 inhibitors may indirectly influence the progression of neurodegenerative pathology.

However, current clinical evidence remains limited, and most data supporting neuroprotective effects derive from experimental models or observational studies in diabetic populations. Future research should therefore focus on well-designed clinical trials in biomarker-defined AD cohorts, as well as mechanistic studies aimed at clarifying the relative contribution of peripheral metabolic correction versus direct central effects. Overall, while SGLT2 inhibitors represent mechanistically plausible and translationally attractive repurposing candidates, further investigation is required to determine their true therapeutic potential in Alzheimer’s disease.

## Data Availability

No new data were created or analyzed in this study. Data sharing is not applicable to this article.
